# Effectiveness of montelukast for uremic pruritus in hemodialysis patients: A protocol for systematic review and meta-analysis

**DOI:** 10.1097/MD.0000000000023229

**Published:** 2020-11-13

**Authors:** Chao-qing Gao, Jia-jun Zhou, Ya-yin Tan, Chang-jun Tong

**Affiliations:** Hemopurification Center, Yijishan Hospital of Wannan Medical College, Wuhu, China.

**Keywords:** hemodialysis, montelukast, protocol, systematic review, uremic pruritus

## Abstract

**Background:**

Uremic pruritus (UP) is a common and tormenting symptom in end-stage renal disease patients undergoing maintenance hemodialysis. An increasing number of studies have been published in recent years to support the effectiveness of montelukast for UP. We will conduct a comprehensive systematic review and meta-analysis to evaluate effectiveness of montelukast for UP in hemodialysis patients.

**Methods:**

The following electronic databases were searched: Pubmed, Embase, Web of Science, Cochrane Library, the China National Knowledge Infrastructure, Chinese Biomedical Literature Database, and China Science and Technology Journal Database. The range of publication time was from the inception of the database to December 2020. Two reviewers will independently conduct article selection, data collection, and assessment of risk of bias. Any disagreement will be resolved by discussion with the third reviewer. Meta-analysis will be performed by Review Manager 5.3. The Cochrane Collaboration tool will be used to assess the risk of bias.

**Results:**

This study will provide a systematic synthesis of current published data to explore the effectiveness of montelukast for UP in hemodialysis patients.

**Conclusions:**

This systematic review and meta-analysis will provide clinical evidence for the effectiveness of montelukast for UP in hemodialysis patients and inform our understanding of the value of montelukast in improving pruritus symptoms. This study will help clinicians, patients, and policy makers to make better decisions regarding the appropriate role of montelukast as a part of patient management routines.

**Study registration number:**

INPLASY2020100043.

## 1 Introduction

Uremic pruritus (UP) is a common and tormenting symptom in end-stage renal disease patients undergoing maintenance hemodialysis.^[1]^ The prevalence of UP in maintenance hemodialysis patients is 22% to 90%.^[2–4]^ It affects patients’ life quality, emotional state, social relations, and increases mortality.^[5,6]^ One study reported that UP was associated with two-year cardiovascular mortality in long term hemodialysis patients, and was 1 of the predictors of 24-month cardiovascular mortality in maintenance hemodialysis patients.^[7]^ The pathogenesis of UP remains obscure. Parathormone and hstamine have been reported as possible mediators of UP. Parathyroidectomy can improve persistent pruritus in some some secondary hyperparathyroidism patients.^[8,9]^ One study showed that plasma histamine levels in pruritic patients undergoing continuous ambulatory peritoneal dialysis were higher than in nonpruritic patients, and during ondansetron treatment, the severity of pruritus and plasma histamine levels were improved significantly.^[10]^

Daily topical emollients such as tacrolimus ointments, gamma linolenic acid ointment should be regarded as baseline therapy.^[11]^ Ointment strongly improved pruritus during treatment period, while pruritus rose back to baseline values within days after end of treatment.^[12]^ Hence the addition of systemic therapy is necessary. It mainly contains μ-opioid receptor antagonists (naltrexone), κ-opioid receptor agonists (nalfurafine), gabapentin, pentoxifylline, thalidomide and so on.^[13–15]^ Treatment has been mainly empirical, and the efficacy of therapies is often insufficient to provide adequate relief of UP in hemodialysis patients.^[16,17]^

Montelukast is a leukotriene receptor antagonist that has been used in asthma, eosinophilic peritonitis, atopic dermatitis and allergic rhinitis.^[18–20]^ Intradermally injected leukotriene B4 could provoke scratching in mice, and high urinary leukotriene E4 levels were connected with itch nightly.^[21]^ It can be seen that leukotriene can cause pruritus. Montelukast can suppress the expression of inflammatory mediators such as substance P that acts as a neurotransmitter in UP.^[22]^ These can explain the antipruritic effect of montelukast for UP. An increasing number of studies have been published in recent years to support the effectiveness of montelukast for UP.^[23,24]^

Up to now, no systematic review and meta-analysis has been performed on the effectiveness of montelukast for UP in hemodialysis patients. In view of this, we will conduct a comprehensive systematic review and meta-analysis to evaluate effectiveness of montelukast for UP in hemodialysis patients.

## 2 Methods

### 2.1 Study registration

This study has been registered on INPLASY (INPLASY2020100043). This systematic review and meta-analysis will be performed under the guide of the Preferred Reporting Items for Systematic Reviews and Meta-Analyses statement checklist.^[25]^

### 2.2 Eligibility criteria

#### 2.2.1 Types of studies

Randomized controlled trials of montelukast for UP in hemodialysis patients will be considered for inclusion without language limitation. The included trials were required to contain statistical methods and accurate data. Duplicate studies, animal experiments, reviews or case reports were excluded.

#### 2.2.2 Types of participants

Hemodialysis patients with UP will be included without restrictions of the nationality, age, gender, and race.

#### 2.2.3 Types of interventions

In the treatment group, patients were given montelukast with no limitations of dosage and duration of intervention. Randomized controlled trials that have control groups with conventional medication treatments or placebo will be included.

#### 2.2.4 Types of outcomes

Pruritus severity as assessed using a visual analog scale, and the Detailed Pruritus Scale will be designated as the primary outcomes. Secondary outcome will included adverse events due to the medication.

### 2.3 Search strategy

The following electronic databases were searched: Pubmed, Embase, Web of Science, Cochrane Library, the China National Knowledge Infrastructure, Chinese Biomedical Literature Database, and China Science and Technology Journal Database. The range of publication time was from the inception of the database to December 2020. The detailed search strategy for PubMed is shown in Table 1. The similar search strategies will be used for other electronic databases.

**Table 1:** Search strategy of PubMed.

Number	Search terms
1	pruritus
2	itch
3	uremic pruritus
4	Or 1–3
5	end-stage renal disease
6	uremia
7	toxuria
8	chronic kidney disease
9	chronic renal failure
10	Or 5–9
11	hemodialysis
12	renal dialysis
13	continuous renal replacement therapy
14	Or 11–13
15	montelukast
16	montelukast sodium
17	leukotriene antagonists
18	Or 15–17
19	Randomized controlled trial
20	Clinical trial
21	Random
22	Randomized
23	Randomly
24	Trial
25	Placebo
26	Or 19–25
27	4 and 10 and 14 and 18 and 26

### 2.4 Selection of studies

All searched articles will be imported into EndNote 7.0 software, and duplicates will be excluded by software. After removing duplicates, 2 reviewers will independently evaluate all the eligible articles for inclusion. Titles and abstracts will be scanned to eliminate all irrelevant records. The remaining records will be read by full texts in further assessing the inclusion of the study. Any disagreement will be resolved by discussion with the third reviewer. A Preferred Reporting Items for Systematic Reviews and Meta-Analyses flowchart will be designed to describe the details of selection process.

### 2.5 Data extraction and management

After selection, 2 reviewers will independently conduct data extraction. Any disagreement will be resolved by discussion with the third reviewer. The following information was extracted independently by reviewers: author's name, publication year, country, title of journal, study design, sample sizes, treatment and control intervention and outcome measures. If some important information is missing, we will contact original authors by email to request detailed information.

### 2.6 Assessment of risk of bias

The Cochrane Collaboration tool will be used to assess the risk of bias of the selected studies. The following aspects were assessed independently by 2 reviews: random sequence generation, allocation concealment, blinding of participants and personnel, blinding of outcome assessment, incomplete outcome data, selective reporting, and other bias. Disagreements were analyzed by the third reviewer.

### 2.7 Data synthesis and analysis

#### 2.7.1 Data synthesis

Data synthesis will be performed by Review Manager 5.3. Continuous outcomes will be used for mean difference with 95% confidence interval. Risk ratio will be used for dichotomous outcomes with 95% confidence interval. Heterogeneity will be examined using the *I*^2^ test. If the *I*^2^ value > 50%, the random effects model will be used. Otherwise, the fixed effects model will be utilized. If significant heterogeneity still exists after subgroup analysis, descriptive summary will be reported.

#### 2.7.2 Subgroup analysis

If included studies have greater heterogeneity, subgroup analysis will be conducted to explore potential sources of heterogeneity. Subgroup analysis will be divided by different participant characteristics, disease course, controls, interventions and outcome measures.

#### 2.7.3 Sensitivity analysis

Sensitivity analysis will be applied to check the robustness and reliability of pooled results made in the review process. We will perform meta-analysis again after deleting low-quality studies and apply different statistical methods.

#### 2.7.4 Reporting bias

If there are enough trials (≥10 trials) for meta-analysis, we will evaluate the reporting bias with funnel plot and Egger regression analysis.^[26,27]^

### 2.8 Ethics and dissemination

The ethics approval is not necessary because the data are extracted from the published literature and they are not related to the individual patient's data. The results of this systematic review and meta-analysis will be published in a peer-reviewed journal.

## 3 Discussion

To our knowledge, this is the first systematic review and meta-analysis to conduct a comprehensive literature search and provide a systematic synthesis of current published data to explore the effectiveness of montelukast for UP in hemodialysis patients. Seven electronic literature databases will be searched to avoid missing any potential eligible studies, and rigorous methodology will be applied to examine studies reporting montelukast for UP in hemodialysis patients. We believe that this systematic review and meta-analysis will provide clinical evidence for the effectiveness of montelukast for UP in hemodialysis patients and inform our understanding of the value of montelukast in improving pruritus symptoms. This study will help clinicians, patients, and policy makers to make better decisions regarding the appropriate role of montelukast as a part of patient management routines.

## Author contributions

**Conceptualization**: Chao-qing Gao, Ya-yin Tan.

**Data curation**: Chao-qing Gao, Jia-jun Zhou.

**Formal analysis**: Jia-jun Zhou, Ya-yin Tan, Chang-jun Tong.

**Investigation**: Jia-jun Zhou, Ya-yin Tan.

**Methodology**: Chao-qing Gao, Jia-jun Zhou, Chang-jun Tong.

**Project administration**: Ya-yin Tan.

**Resources**: Chao-qing Gao, Jia-jun Zhou, Ya-yin Tan.

**Software**: Jia-jun Zhou, Chang-jun Tong.

**Supervision**: Chao-qing Gao, Jia-jun Zhou, Ya-yin Tan.

**Validation:** Ya-yin Tan.

**Visualization**: Chao-qing Gao, Jia-jun Zhou, Chang-jun Tong.

**Writing – original draft**: Chao-qing Gao, Jia-jun Zhou.

**Writing – review & editing**: Chao-qing Gao, Jia-jun Zhou, Ya-yin Tan, Chang-jun Tong.
